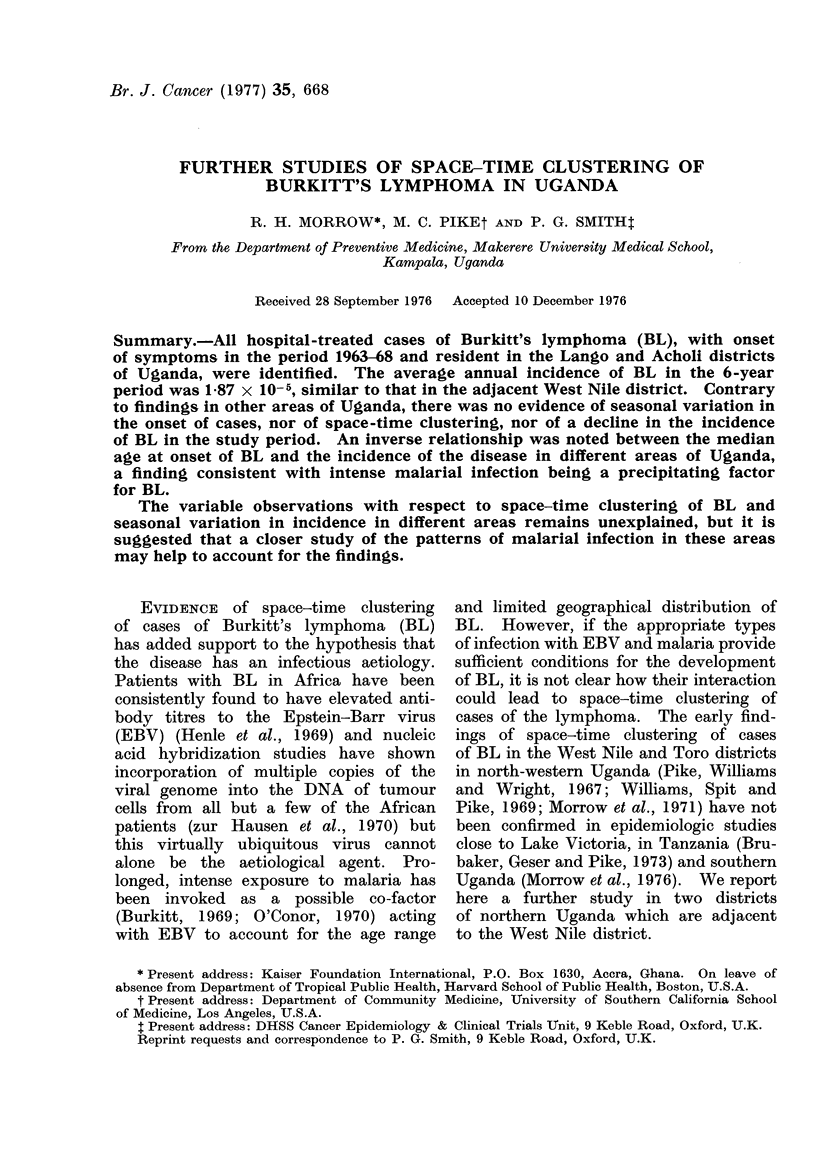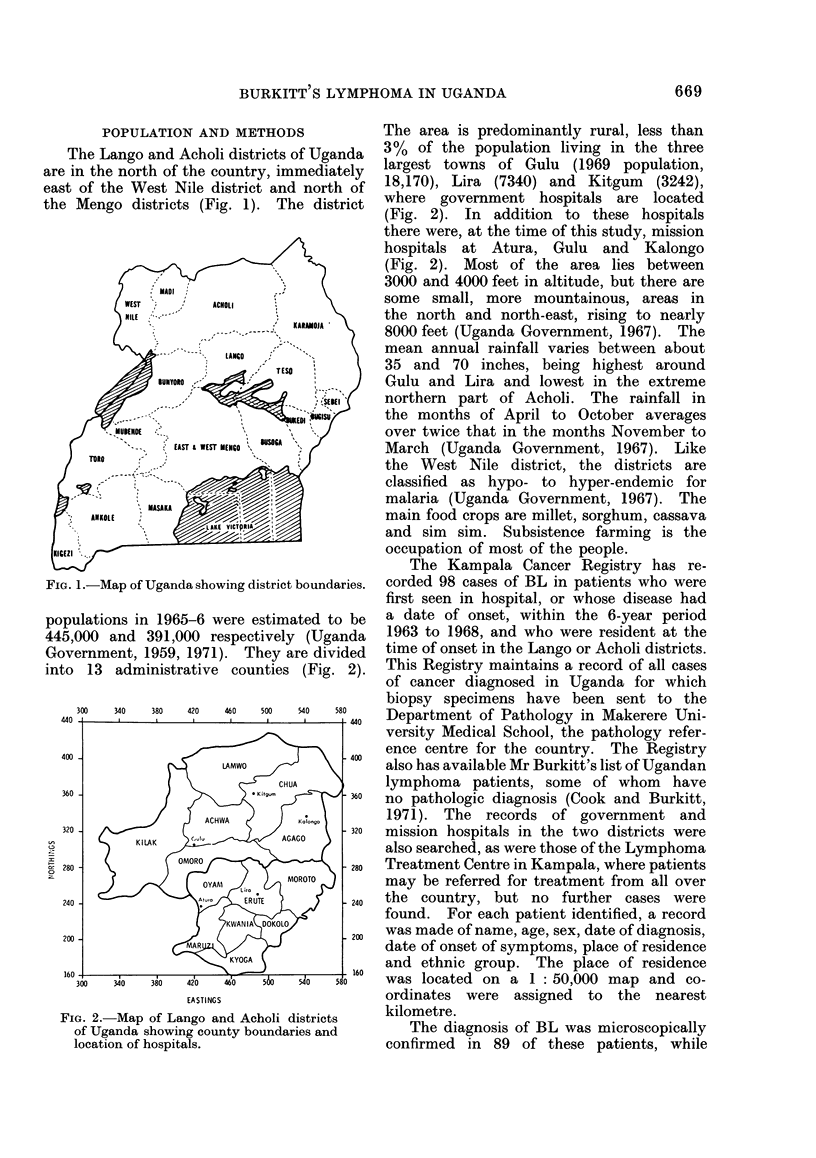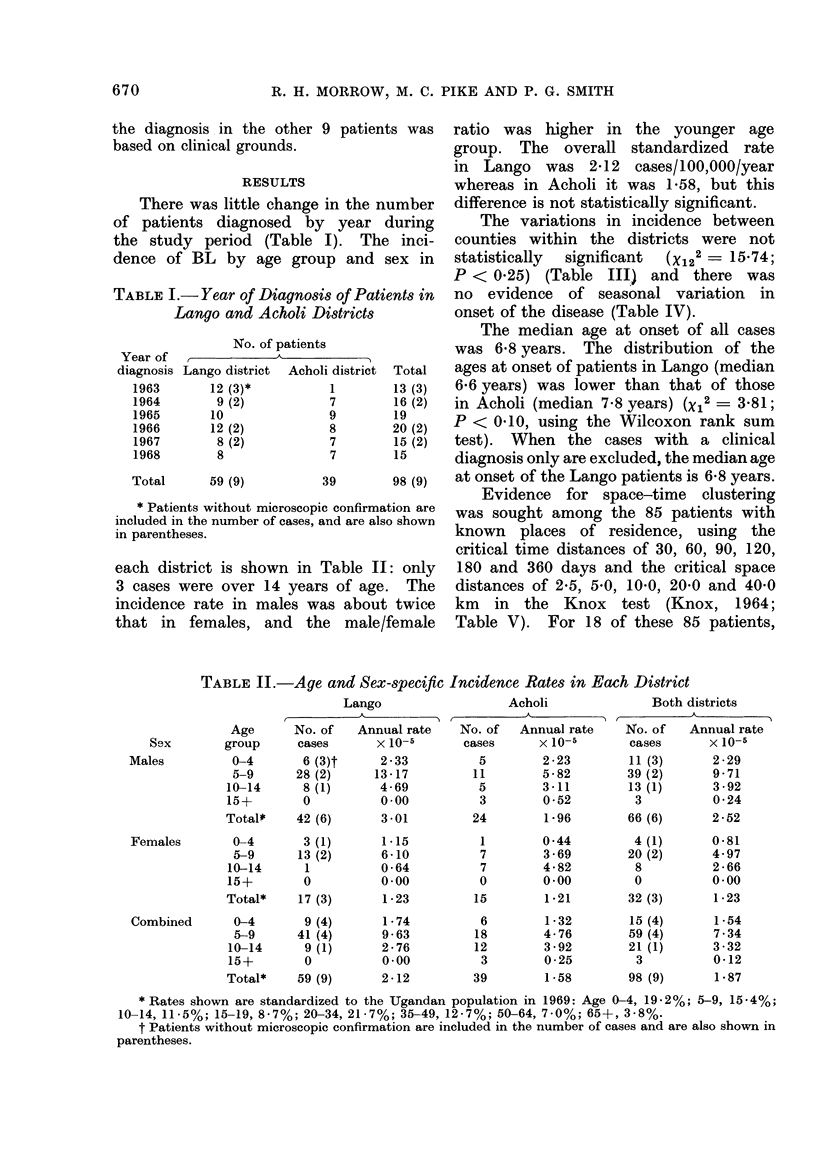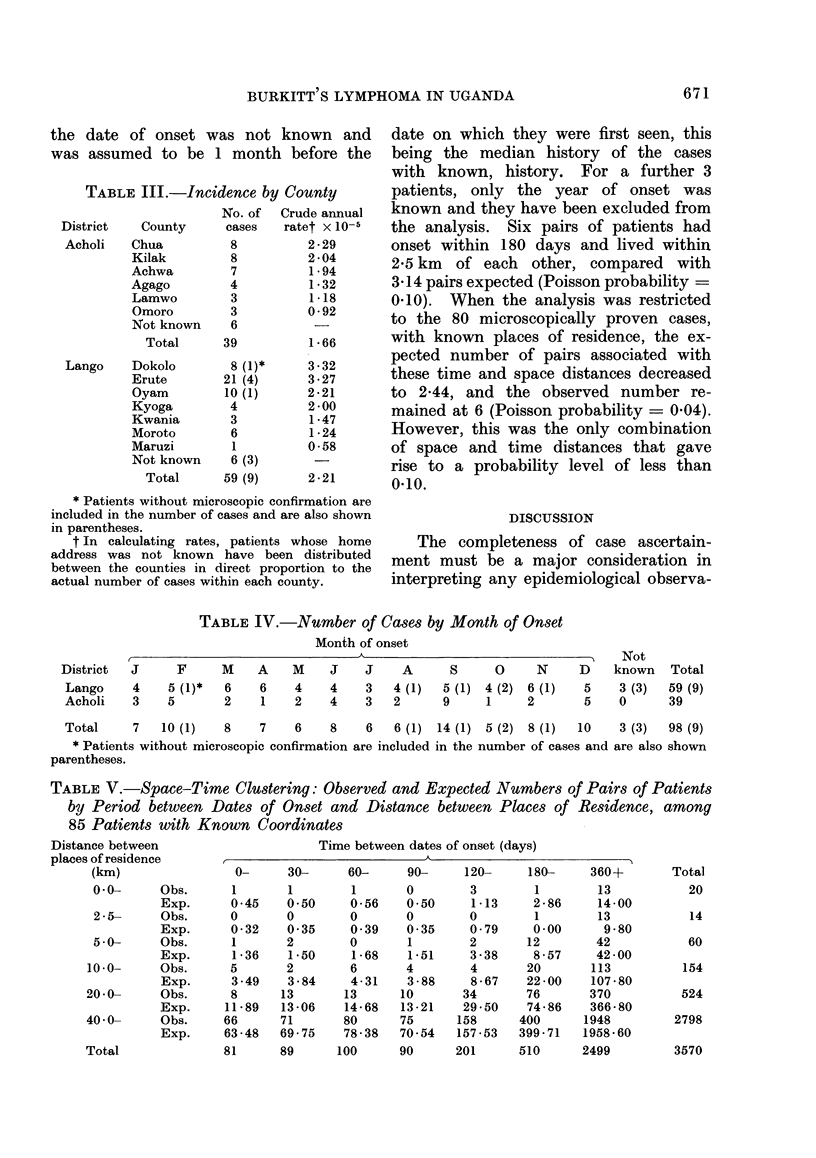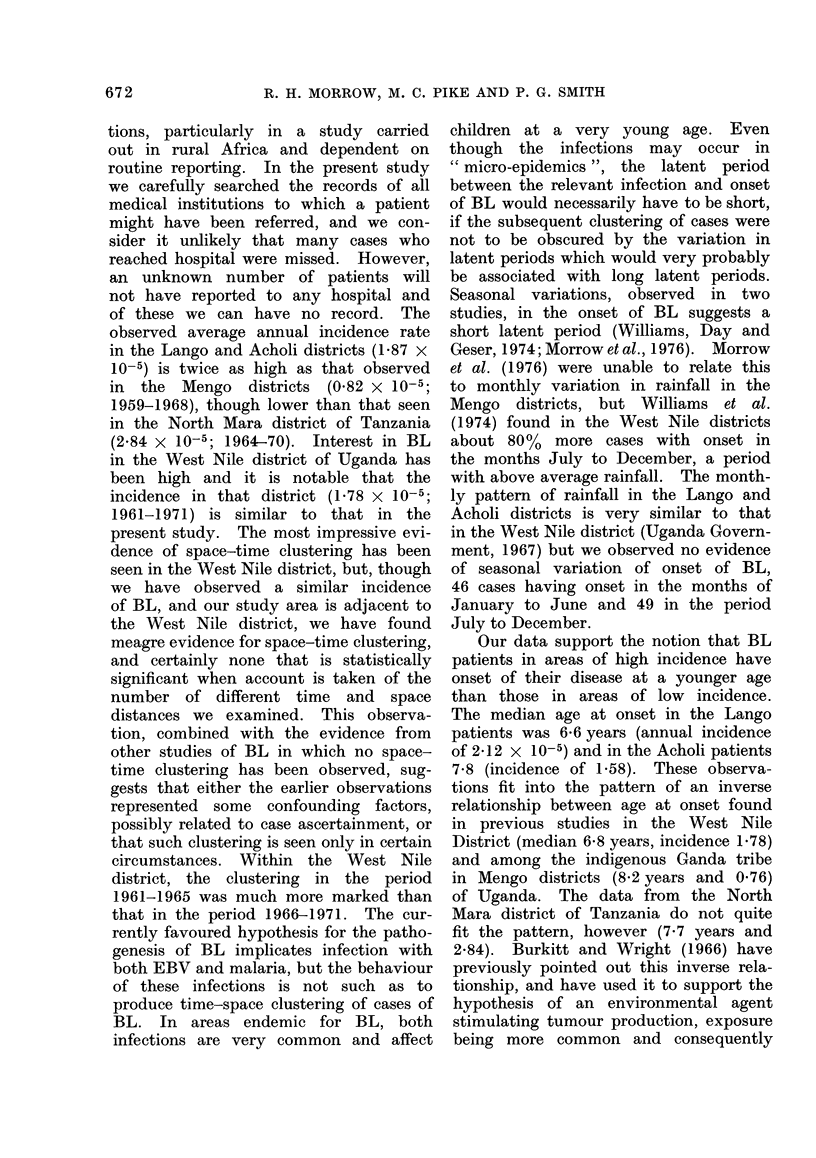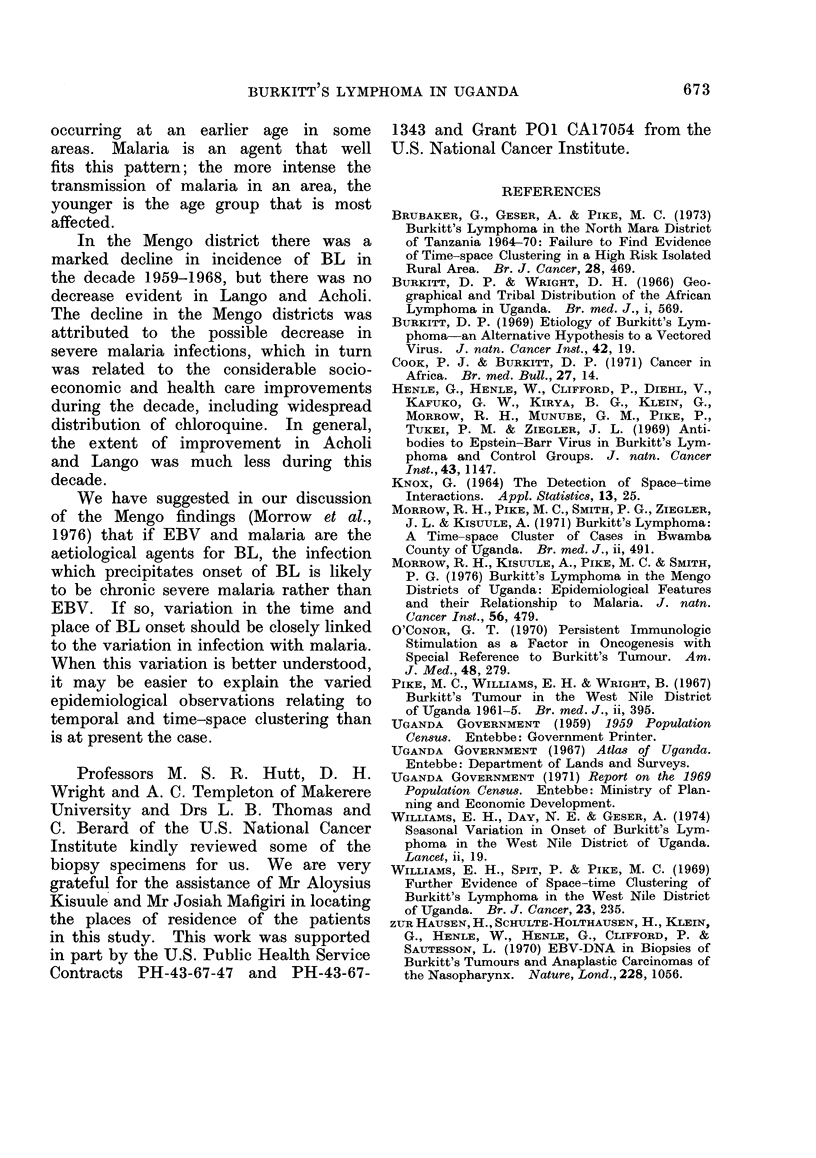# Further studies of space-time clustering of Burkitt's lymphoma in Uganda.

**DOI:** 10.1038/bjc.1977.102

**Published:** 1977-05

**Authors:** R. H. Morrow, M. C. Pike, P. G. Smith

## Abstract

All hospital-treated cases of Burkitt's lymphoma (BL), with onset of symptoms in the period 1963-68 and resident in the Lango and Acholi districts of Uganda, were identified. The average annual incidence of BL in the 6-year period was 1-87 X 10(-5), similar to that in the adjacent West Nile district. Contrary to findings in other areas of Uganda, there was no evidence of seasonal variation in the onset of cases, nor of space-time clustering, nor of a decline in the incidence of BL in the study period. An inverse relationship was noted between the median age at onset of BL and the incidence of the disease in different areas of Uganda, a finding consistent with intense malarial infection being a precipitating factor for BL. The variable observations with respect to space-time clustering of BL and seasonal variation in incidence in different areas remains unexplained, but it is suggested that a closer study of the patterns of malarial infection in these areas may help to account for the findings.


*Br. J. Cancer* (1977) **35**, 668

### FURTHER STUDIES OF SPACE–TIME CLUSTERING OF BURKITT'S LYMPHOMA IN UGANDA

R. H. MORROW*, M. C. PIKE† AND P. G. SMITH‡

*From the Department of Preventive Medicine, Makerere University Medical School, Kampala, Uganda*

Received 28 September 1976 Accepted 10 December 1976

**Summary.—All hospital-treated cases of Burkitt's lymphoma (BL), with onset of symptoms in the period 1963–68 and resident in the Lango and Acholi districts of Uganda, were identified. The average annual incidence of BL in the 6-year period was $1{\cdot}87 \times 10^{-5}$, similar to that in the adjacent West Nile district. Contrary to findings in other areas of Uganda, there was no evidence of seasonal variation in the onset of cases, nor of space-time clustering, nor of a decline in the incidence of BL in the study period. An inverse relationship was noted between the median age at onset of BL and the incidence of the disease in different areas of Uganda, a finding consistent with intense malarial infection being a precipitating factor for BL.**

**The variable observations with respect to space–time clustering of BL and seasonal variation in incidence in different areas remains unexplained, but it is suggested that a closer study of the patterns of malarial infection in these areas may help to account for the findings.**

EVIDENCE of space–time clustering of cases of Burkitt's lymphoma (BL) has added support to the hypothesis that the disease has an infectious aetiology. Patients with BL in Africa have been consistently found to have elevated antibody titres to the Epstein–Barr virus (EBV) (Henle *et al.*, 1969) and nucleic acid hybridization studies have shown incorporation of multiple copies of the viral genome into the DNA of tumour cells from all but a few of the African patients (zur Hausen *et al.*, 1970) but this virtually ubiquitous virus cannot alone be the aetiological agent. Prolonged, intense exposure to malaria has been invoked as a possible co-factor (Burkitt, 1969; O'Conor, 1970) acting with EBV to account for the age range and limited geographical distribution of BL. However, if the appropriate types of infection with EBV and malaria provide sufficient conditions for the development of BL, it is not clear how their interaction could lead to space–time clustering of cases of the lymphoma. The early findings of space–time clustering of cases of BL in the West Nile and Toro districts in north-western Uganda (Pike, Williams and Wright, 1967; Williams, Spit and Pike, 1969; Morrow *et al.*, 1971) have not been confirmed in epidemiologic studies close to Lake Victoria, in Tanzania (Brubaker, Geser and Pike, 1973) and southern Uganda (Morrow *et al.*, 1976). We report here a further study in two districts of northern Uganda which are adjacent to the West Nile district.

* Present address: Kaiser Foundation International, P.O. Box 1630, Accra, Ghana. On leave of absence from Department of Tropical Public Health, Harvard School of Public Health, Boston, U.S.A.

† Present address: Department of Community Medicine, University of Southern California School of Medicine, Los Angeles, U.S.A.

‡ Present address: DHSS Cancer Epidemiology & Clinical Trials Unit, 9 Keble Road, Oxford, U.K.

Reprint requests and correspondence to P. G. Smith, 9 Keble Road, Oxford, U.K.

### POPULATION AND METHODS

The Lango and Acholi districts of Uganda are in the north of the country, immediately east of the West Nile district and north of the Mengo districts (Fig. 1). The district populations in 1965–6 were estimated to be 445,000 and 391,000 respectively (Uganda Government, 1959, 1971). They are divided into 13 administrative counties (Fig. 2).

FIG. 1.—Map of Uganda showing district boundaries.

FIG. 2.—Map of Lango and Acholi districts of Uganda showing county boundaries and location of hospitals.

The area is predominantly rural, less than 3% of the population living in the three largest towns of Gulu (1969 population, 18,170), Lira (7340) and Kitgum (3242), where government hospitals are located (Fig. 2). In addition to these hospitals there were, at the time of this study, mission hospitals at Atura, Gulu and Kalongo (Fig. 2). Most of the area lies between 3000 and 4000 feet in altitude, but there are some small, more mountainous, areas in the north and north-east, rising to nearly 8000 feet (Uganda Government, 1967). The mean annual rainfall varies between about 35 and 70 inches, being highest around Gulu and Lira and lowest in the extreme northern part of Acholi. The rainfall in the months of April to October averages over twice that in the months November to March (Uganda Government, 1967). Like the West Nile district, the districts are classified as hypo- to hyper-endemic for malaria (Uganda Government, 1967). The main food crops are millet, sorghum, cassava and sim sim. Subsistence farming is the occupation of most of the people.

The Kampala Cancer Registry has recorded 98 cases of BL in patients who were first seen in hospital, or whose disease had a date of onset, within the 6-year period 1963 to 1968, and who were resident at the time of onset in the Lango or Acholi districts. This Registry maintains a record of all cases of cancer diagnosed in Uganda for which biopsy specimens have been sent to the Department of Pathology in Makerere University Medical School, the pathology reference centre for the country. The Registry also has available Mr Burkitt's list of Ugandan lymphoma patients, some of whom have no pathologic diagnosis (Cook and Burkitt, 1971). The records of government and mission hospitals in the two districts were also searched, as were those of the Lymphoma Treatment Centre in Kampala, where patients may be referred for treatment from all over the country, but no further cases were found. For each patient identified, a record was made of name, age, sex, date of diagnosis, date of onset of symptoms, place of residence and ethnic group. The place of residence was located on a 1 : 50,000 map and coordinates were assigned to the nearest kilometre.

The diagnosis of BL was microscopically confirmed in 89 of these patients, while the diagnosis in the other 9 patients was based on clinical grounds.

### RESULTS

There was little change in the number of patients diagnosed by year during the study period (Table I). The incidence of BL by age group and sex in each district is shown in Table II: only 3 cases were over 14 years of age. The incidence rate in males was about twice that in females, and the male/female ratio was higher in the younger age group. The overall standardized rate in Lango was 2·12 cases/100,000/year whereas in Acholi it was 1·58, but this difference is not statistically significant.

TABLE I.—*Year of Diagnosis of Patients in Lango and Acholi Districts*

| Year of diagnosis | No. of patients: Lango district | Acholi district | Total |
|---|---|---|---|
| 1963 | 12 (3)* | 1 | 13 (3) |
| 1964 | 9 (2) | 7 | 16 (2) |
| 1965 | 10 | 9 | 19 |
| 1966 | 12 (2) | 8 | 20 (2) |
| 1967 | 8 (2) | 7 | 15 (2) |
| 1968 | 8 | 7 | 15 |
| Total | 59 (9) | 39 | 98 (9) |

* Patients without microscopic confirmation are included in the number of cases, and are also shown in parentheses.

The variations in incidence between counties within the districts were not statistically significant ($\chi_{12}^2 = 15{\cdot}74$; $P < 0{\cdot}25$) (Table III) and there was no evidence of seasonal variation in onset of the disease (Table IV).

The median age at onset of all cases was 6·8 years. The distribution of the ages at onset of patients in Lango (median 6·6 years) was lower than that of those in Acholi (median 7·8 years) ($\chi_1^2 = 3{\cdot}81$; $P < 0{\cdot}10$, using the Wilcoxon rank sum test). When the cases with a clinical diagnosis only are excluded, the median age at onset of the Lango patients is 6·8 years.

Evidence for space–time clustering was sought among the 85 patients with known places of residence, using the critical time distances of 30, 60, 90, 120, 180 and 360 days and the critical space distances of 2·5, 5·0, 10·0, 20·0 and 40·0 km in the Knox test (Knox, 1964; Table V). For 18 of these 85 patients,

TABLE II.—*Age and Sex-specific Incidence Rates in Each District*

| Sex | Age group | Lango: No. of cases | Lango: Annual rate ×10⁻⁵ | Acholi: No. of cases | Acholi: Annual rate ×10⁻⁵ | Both districts: No. of cases | Both districts: Annual rate ×10⁻⁵ |
|---|---|---|---|---|---|---|---|
| Males | 0–4 | 6 (3)† | 2·33 | 5 | 2·23 | 11 (3) | 2·29 |
| | 5–9 | 28 (2) | 13·17 | 11 | 5·82 | 39 (2) | 9·71 |
| | 10–14 | 8 (1) | 4·69 | 5 | 3·11 | 13 (1) | 3·92 |
| | 15+ | 0 | 0·00 | 3 | 0·52 | 3 | 0·24 |
| | Total* | 42 (6) | 3·01 | 24 | 1·96 | 66 (6) | 2·52 |
| Females | 0–4 | 3 (1) | 1·15 | 1 | 0·44 | 4 (1) | 0·81 |
| | 5–9 | 13 (2) | 6·10 | 7 | 3·69 | 20 (2) | 4·97 |
| | 10–14 | 1 | 0·64 | 7 | 4·82 | 8 | 2·66 |
| | 15+ | 0 | 0·00 | 0 | 0·00 | 0 | 0·00 |
| | Total* | 17 (3) | 1·23 | 15 | 1·21 | 32 (3) | 1·23 |
| Combined | 0–4 | 9 (4) | 1·74 | 6 | 1·32 | 15 (4) | 1·54 |
| | 5–9 | 41 (4) | 9·63 | 18 | 4·76 | 59 (4) | 7·34 |
| | 10–14 | 9 (1) | 2·76 | 12 | 3·92 | 21 (1) | 3·32 |
| | 15+ | 0 | 0·00 | 3 | 0·25 | 3 | 0·12 |
| | Total* | 59 (9) | 2·12 | 39 | 1·58 | 98 (9) | 1·87 |

* Rates shown are standardized to the Ugandan population in 1969: Age 0–4, 19·2%; 5–9, 15·4%; 10–14, 11·5%; 15–19, 8·7%; 20–34, 21·7%; 35–49, 12·7%; 50–64, 7·0%; 65+, 3·8%.

† Patients without microscopic confirmation are included in the number of cases and are also shown in parentheses.

the date of onset was not known and was assumed to be 1 month before the date on which they were first seen, this being the median history of the cases with known, history. For a further 3 patients, only the year of onset was known and they have been excluded from the analysis. Six pairs of patients had onset within 180 days and lived within 2·5 km of each other, compared with 3·14 pairs expected (Poisson probability = 0·10). When the analysis was restricted to the 80 microscopically proven cases, with known places of residence, the expected number of pairs associated with these time and space distances decreased to 2·44, and the observed number remained at 6 (Poisson probability = 0·04). However, this was the only combination of space and time distances that gave rise to a probability level of less than 0·10.

TABLE III.—*Incidence by County*

| District | County | No. of cases | Crude annual rate† $\times 10^{-5}$ |
|---|---|---|---|
| Acholi | Chua | 8 | 2·29 |
| | Kilak | 8 | 2·04 |
| | Achwa | 7 | 1·94 |
| | Agago | 4 | 1·32 |
| | Lamwo | 3 | 1·18 |
| | Omoro | 3 | 0·92 |
| | Not known | 6 | — |
| | Total | 39 | 1·66 |
| Lango | Dokolo | 8 (1)* | 3·32 |
| | Erute | 21 (4) | 3·27 |
| | Oyam | 10 (1) | 2·21 |
| | Kyoga | 4 | 2·00 |
| | Kwania | 3 | 1·47 |
| | Moroto | 6 | 1·24 |
| | Maruzi | 1 | 0·58 |
| | Not known | 6 (3) | — |
| | Total | 59 (9) | 2·21 |

* Patients without microscopic confirmation are included in the number of cases and are also shown in parentheses.

† In calculating rates, patients whose home address was not known have been distributed between the counties in direct proportion to the actual number of cases within each county.

DISCUSSION

The completeness of case ascertainment must be a major consideration in interpreting any epidemiological observa-

TABLE IV.—*Number of Cases by Month of Onset*

| District | Month of onset J | F | M | A | M | J | J | A | S | O | N | D | Not known | Total |
|---|---|---|---|---|---|---|---|---|---|---|---|---|---|---|
| Lango | 4 | 5 (1)* | 6 | 6 | 4 | 4 | 3 | 4 (1) | 5 (1) | 4 (2) | 6 (1) | 5 | 3 (3) | 59 (9) |
| Acholi | 3 | 5 | 2 | 1 | 2 | 4 | 3 | 2 | 9 | 1 | 2 | 5 | 0 | 39 |
| Total | 7 | 10 (1) | 8 | 7 | 6 | 8 | 6 | 6 (1) | 14 (1) | 5 (2) | 8 (1) | 10 | 3 (3) | 98 (9) |

* Patients without microscopic confirmation are included in the number of cases and are also shown parentheses.

TABLE V.—*Space–Time Clustering: Observed and Expected Numbers of Pairs of Patients by Period between Dates of Onset and Distance between Places of Residence, among 85 Patients with Known Coordinates*

| Distance between places of residence (km) | | Time between dates of onset (days) 0– | 30– | 60– | 90– | 120– | 180– | 360+ | Total |
|---|---|---|---|---|---|---|---|---|---|
| 0·0– | Obs. | 1 | 1 | 1 | 0 | 3 | 1 | 13 | 20 |
| | Exp. | 0·45 | 0·50 | 0·56 | 0·50 | 1·13 | 2·86 | 14·00 | |
| 2·5– | Obs. | 0 | 0 | 0 | 0 | 0 | 1 | 13 | 14 |
| | Exp. | 0·32 | 0·35 | 0·39 | 0·35 | 0·79 | 0·00 | 9·80 | |
| 5·0– | Obs. | 1 | 2 | 0 | 1 | 2 | 12 | 42 | 60 |
| | Exp. | 1·36 | 1·50 | 1·68 | 1·51 | 3·38 | 8·57 | 42·00 | |
| 10·0– | Obs. | 5 | 2 | 6 | 4 | 4 | 20 | 113 | 154 |
| | Exp. | 3·49 | 3·84 | 4·31 | 3·88 | 8·67 | 22·00 | 107·80 | |
| 20·0– | Obs. | 8 | 13 | 13 | 10 | 34 | 76 | 370 | 524 |
| | Exp. | 11·89 | 13·06 | 14·68 | 13·21 | 29·50 | 74·86 | 366·80 | |
| 40·0– | Obs. | 66 | 71 | 80 | 75 | 158 | 400 | 1948 | 2798 |
| | Exp. | 63·48 | 69·75 | 78·38 | 70·54 | 157·53 | 399·71 | 1958·60 | |
| Total | | 81 | 89 | 100 | 90 | 201 | 510 | 2499 | 3570 |

tions, particularly in a study carried out in rural Africa and dependent on routine reporting. In the present study we carefully searched the records of all medical institutions to which a patient might have been referred, and we consider it unlikely that many cases who reached hospital were missed. However, an unknown number of patients will not have reported to any hospital and of these we can have no record. The observed average annual incidence rate in the Lango and Acholi districts ($1{\cdot}87 \times 10^{-5}$) is twice as high as that observed in the Mengo districts ($0{\cdot}82 \times 10^{-5}$; 1959–1968), though lower than that seen in the North Mara district of Tanzania ($2{\cdot}84 \times 10^{-5}$; 1964–70). Interest in BL in the West Nile district of Uganda has been high and it is notable that the incidence in that district ($1{\cdot}78 \times 10^{-5}$; 1961–1971) is similar to that in the present study. The most impressive evidence of space–time clustering has been seen in the West Nile district, but, though we have observed a similar incidence of BL, and our study area is adjacent to the West Nile district, we have found meagre evidence for space–time clustering, and certainly none that is statistically significant when account is taken of the number of different time and space distances we examined. This observation, combined with the evidence from other studies of BL in which no space–time clustering has been observed, suggests that either the earlier observations represented some confounding factors, possibly related to case ascertainment, or that such clustering is seen only in certain circumstances. Within the West Nile district, the clustering in the period 1961–1965 was much more marked than that in the period 1966–1971. The currently favoured hypothesis for the pathogenesis of BL implicates infection with both EBV and malaria, but the behaviour of these infections is not such as to produce time–space clustering of cases of BL. In areas endemic for BL, both infections are very common and affect children at a very young age. Even though the infections may occur in " micro-epidemics ", the latent period between the relevant infection and onset of BL would necessarily have to be short, if the subsequent clustering of cases were not to be obscured by the variation in latent periods which would very probably be associated with long latent periods. Seasonal variations, observed in two studies, in the onset of BL suggests a short latent period (Williams, Day and Geser, 1974; Morrow *et al.*, 1976). Morrow *et al.* (1976) were unable to relate this to monthly variation in rainfall in the Mengo districts, but Williams *et al.* (1974) found in the West Nile districts about 80% more cases with onset in the months July to December, a period with above average rainfall. The monthly pattern of rainfall in the Lango and Acholi districts is very similar to that in the West Nile district (Uganda Government, 1967) but we observed no evidence of seasonal variation of onset of BL, 46 cases having onset in the months of January to June and 49 in the period July to December.

Our data support the notion that BL patients in areas of high incidence have onset of their disease at a younger age than those in areas of low incidence. The median age at onset in the Lango patients was 6·6 years (annual incidence of $2{\cdot}12 \times 10^{-5}$) and in the Acholi patients 7·8 (incidence of 1·58). These observations fit into the pattern of an inverse relationship between age at onset found in previous studies in the West Nile District (median 6·8 years, incidence 1·78) and among the indigenous Ganda tribe in Mengo districts (8·2 years and 0·76) of Uganda. The data from the North Mara district of Tanzania do not quite fit the pattern, however (7·7 years and 2·84). Burkitt and Wright (1966) have previously pointed out this inverse relationship, and have used it to support the hypothesis of an environmental agent stimulating tumour production, exposure being more common and consequently occurring at an earlier age in some areas. Malaria is an agent that well fits this pattern; the more intense the transmission of malaria in an area, the younger is the age group that is most affected.

In the Mengo district there was a marked decline in incidence of BL in the decade 1959–1968, but there was no decrease evident in Lango and Acholi. The decline in the Mengo districts was attributed to the possible decrease in severe malaria infections, which in turn was related to the considerable socio-economic and health care improvements during the decade, including widespread distribution of chloroquine. In general, the extent of improvement in Acholi and Lango was much less during this decade.

We have suggested in our discussion of the Mengo findings (Morrow *et al.*, 1976) that if EBV and malaria are the aetiological agents for BL, the infection which precipitates onset of BL is likely to be chronic severe malaria rather than EBV. If so, variation in the time and place of BL onset should be closely linked to the variation in infection with malaria. When this variation is better understood, it may be easier to explain the varied epidemiological observations relating to temporal and time-space clustering than is at present the case.

Professors M. S. R. Hutt, D. H. Wright and A. C. Templeton of Makerere University and Drs L. B. Thomas and C. Berard of the U.S. National Cancer Institute kindly reviewed some of the biopsy specimens for us. We are very grateful for the assistance of Mr Aloysius Kisuule and Mr Josiah Mafigiri in locating the places of residence of the patients in this study. This work was supported in part by the U.S. Public Health Service Contracts PH-43-67-47 and PH-43-67-1343 and Grant P01 CA17054 from the U.S. National Cancer Institute.

### REFERENCES

Brubaker, G., Geser, A. & Pike, M. C. (1973) Burkitt's Lymphoma in the North Mara District of Tanzania 1964–70: Failure to Find Evidence of Time-space Clustering in a High Risk Isolated Rural Area. *Br. J. Cancer*, **28**, 469.

Burkitt, D. P. & Wright, D. H. (1966) Geographical and Tribal Distribution of the African Lymphoma in Uganda. *Br. med. J.*, i, 569.

Burkitt, D. P. (1969) Etiology of Burkitt's Lymphoma—an Alternative Hypothesis to a Vectored Virus. *J. natn. Cancer Inst.*, **42**, 19.

Cook, P. J. & Burkitt, D. P. (1971) Cancer in Africa. *Br. med. Bull.*, **27**, 14.

Henle, G., Henle, W., Clifford, P., Diehl, V., Kafuko, G. W., Kirya, B. G., Klein, G., Morrow, R. H., Munube, G. M., Pike, P., Tukei, P. M. & Ziegler, J. L. (1969) Antibodies to Epstein–Barr Virus in Burkitt's Lymphoma and Control Groups. *J. natn. Cancer Inst.*, **43**, 1147.

Knox, G. (1964) The Detection of Space–time Interactions. *Appl. Statistics*, **13**, 25.

Morrow, R. H., Pike, M. C., Smith, P. G., Ziegler, J. L. & Kisuule, A. (1971) Burkitt's Lymphoma: A Time-space Cluster of Cases in Bwamba County of Uganda. *Br. med. J.*, ii, 491.

Morrow, R. H., Kisuule, A., Pike, M. C. & Smith, P. G. (1976) Burkitt's Lymphoma in the Mengo Districts of Uganda: Epidemiological Features and their Relationship to Malaria. *J. natn. Cancer Inst.*, **56**, 479.

O'Conor, G. T. (1970) Persistent Immunologic Stimulation as a Factor in Oncogenesis with Special Reference to Burkitt's Tumour. *Am. J. Med.*, **48**, 279.

Pike, M. C., Williams, E. H. & Wright, B. (1967) Burkitt's Tumour in the West Nile District of Uganda 1961–5. *Br. med. J.*, ii, 395.

Uganda Government (1959) *1959 Population Census*. Entebbe: Government Printer.

Uganda Government (1967) *Atlas of Uganda*. Entebbe: Department of Lands and Surveys.

Uganda Government (1971) *Report on the 1969 Population Census*. Entebbe: Ministry of Planning and Economic Development.

Williams, E. H., Day, N. E. & Geser, A. (1974) Seasonal Variation in Onset of Burkitt's Lymphoma in the West Nile District of Uganda. *Lancet*, ii, 19.

Williams, E. H., Spit, P. & Pike, M. C. (1969) Further Evidence of Space-time Clustering of Burkitt's Lymphoma in the West Nile District of Uganda. *Br. J. Cancer*, **23**, 235.

zur Hausen, H., Schulte-Holthausen, H., Klein, G., Henle, W., Henle, G., Clifford, P. & Sautesson, L. (1970) EBV-DNA in Biopsies of Burkitt's Tumours and Anaplastic Carcinomas of the Nasopharynx. *Nature, Lond.*, **228**, 1056.